# Supplementary material for: Most cancers carry a substantial deleterious load due to Hill-Robertson interference
Source: eLife. 2022 Sep 1;11:e67790. doi: 10.7554/eLife.67790 (PMC9499534; doi:10.7554/eLife.67790)
Supplement: Supplementary file 1. [file elife-67790-supp1.docx]

| Assumption | Anticipated EFFECT ON CONCLUSIONS | Refs |
| --- | --- | --- |
| Exponential DFE for drivers & passengers | ABC estimates effective selection coefficients | Good, 2012. |
| Cells are well-mixed (no spatial structure) | Reduced Hill-Robertson interference | Sottoriva, 2015; Korolev, 2012; Martens 2012; |
| Gompertzian growth dynamics in-between drivers | Decreased inferred strength of drivers relative to no growth constraints | McFarland, 2014. |
| Only 50% of tumors progress to cancer | Mutational burdens widen as progression probability declines | McFarland, 2014. |
| No (reciprocal) sign epistasis | Stronger fitness benefits of drivers in adaptive contexts | Krug, 2014; Rogers 2018 |
| Constant mutation rate for each tumor | Hill-Robertson interference would increase | Goyal, 2012. |
| Simulated tumor is genotyped at transformation | Late (subclonal) mutations are ignored; incidence age reduced | Sottoriva, 2015. |
| Malignancy occurs at 1,000,000 (stem) cells | Reduced variation in cancer incidence times (as true detection times varies) | McFarland, 2014. |
| Subclonal mutations are undetected by genotyping | Lower estimated fitness effects of drivers & passengers (subclonal mutations experience less selection) | McVean, 2000. |
| No dominance | Nearly-unbiased estimate of heterozygous passenger fitness cost; underestimation of driver benefit | Whitlock, 2003. |

**Supplementary File 1. Assumptions of model of tumor evolution and anticipated effects.**
