# Supplementary material for: Most cancers carry a substantial deleterious load due to Hill-Robertson interference
Source: eLife. 2022 Sep 1;11:e67790. doi: 10.7554/eLife.67790 (PMC9499534; doi:10.7554/eLife.67790)
Supplement: Supplementary file 2. [file elife-67790-supp2.docx]

| Broad Category (n) | GDC tumor subtypes in group |
| --- | --- |
| Circulatory (175) | LAML, DLBC |
| Endocrine (899) | ACC, THYM, THCA, PCPG |
| Urinary (1128) | BLCA, KICH, KIRC, KIRP |
| Nervous (928) | LGG, GBM |
| Reproductive (2964) | BRCA, CESC, OV, PRAD, UCEC, TGCT, UCS |
| Respiratory (1558) | LUSC, LUAD, HNSC |
| Skeletal (320) | SARC, MESO |
| Digestive (1762) | PAAD, STAD, READ, CHOL, COAD, ESCA, LIHC |
| Skin (547) | UVM, SKCM |

**Supplementary File 2.** Broad (meta-categories) of cancer groupings used in Figure 2 and Figure 2—figure supplement 12-13.
